# The Contribution of Case Mix, Skill Mix and Care Processes to the Outcomes of Community Hospitals: A Population-Based Observational Study

**DOI:** 10.5334/ijic.5566

**Published:** 2021-06-21

**Authors:** Davide Pianori, Kadjo Yves Cedric Adja, Jacopo Lenzi, Giulia Pieri, Andrea Rossi, Chiara Reno, Maria Pia Fantini

**Affiliations:** 1 Department of Biomedical and Neuromotor Sciences, Alma Mater Studiorum, University of Bologna. Via San Giacomo 12, 40126, Bologna, Italy; 2 Azienda Usl di Imola. Viale Amendola 2, 40026, Imola, Italy

**Keywords:** community hospital, case mix, skill mix, integrated care, frailty, intermediate care, health care services

## Abstract

**Introduction:**

Community hospitals (CHs) could address the emerging complex care needs of patients. We investigated which characteristics of patients’ and CHs affect patient outcomes, in order to identify who could benefit the most from CH care and the best skill mix to deliver this care.

**Methods:**

We analysed all elderly patients discharged from the CHs of Emilia-Romagna, Italy. CH skill mix and care processes were collected with an ad hoc survey. The primary outcome was improvement in the Barthel index (BI) on discharge. Hierarchical regression analysis was performed to test the associations under study.

**Results:**

53% of the patients had a BI improvement ≥10. After adjusting for the diverse case mix of the patients, no significant association was found between CH characteristics and BI improvement. Patient characteristics explained only a portion of the variability in CH performance.

**Discussion:**

Heterogeneity in case mix reflects the nature of CHs, which play context-specific roles as integrators between primary care services and hospitals. Residual variability in BI improvement rates across CHs might be attributed to aspects of care not detected in our survey.

**Conclusions:**

More research is needed to study the impact of CH skill mix and care processes on patient outcomes.

## Introduction

Due to longer life expectancy and lower birth rates, currently all Western countries are facing the aging of populations and the increase of old-age dependency ratio. Older adults are increasingly facing disability, multimorbidity and chronic illnesses, such as diabetes, hypertension, and dementia [1], which indicates a shift from acute to chronic diseases [2].

It is widely acknowledged that this shift requires a profound change in organizational models and practices in order to address and overcome care fragmentation to better co-ordinate care around people’s needs across the care continuum—this is what we might refer to as integrated care [3,4]. It covers a continuum of inter-sectoral and interdisciplinary services and can be distinguished as organizational, service and clinical integration. However, the implementation of integrated care proves to be challenging, turning out difficult in a system not designed with integration in mind [5]. Most health systems are not yet sufficiently equipped with organizational tools and models to face the challenges raised by these new complex care needs and to address concerns about care fragmentation [6]. A range of services and models are being developed worldwide [7,8,9,10,11], but integration of care is highly context-dependent and can be pursued through a wide range of solutions with variable applicability in specific local conditions [3].

Among the possible solutions, intermediate care is an example of integration between acute and primary care. It encompasses a broad spectrum of services, particularly for elderly and frail patients who have complex support needs, In particular, intermediate care services have both “preventive” aims (avoiding unnecessary hospitalizations and “delayed discharges”) and “rehabilitation” aims (supporting discharge and access to rehabilitation services to enforce care close to home); they can reduce pressure on hospitals on one side and help people be as independent as possible after hospitalization on the other side [12,13]. Intermediate care services can be provided in various settings. One of these is represented by community hospitals (CHs), a bed-based intermediate care service defined as hospitals staffed mainly by general practitioners (GPs) and nurses to provide care in a hospital setting. The notion of CH has evolved over time, with a diversity of service-delivery models developing in response to the needs of the local populations and in the context of a broader change in the nature of the delivery of health care services themselves [14]. Overall, CHs play an integrative role in local service provision, by bringing together community, primary and secondary care and possibly supplying a variety of services, such as for example inpatient, outpatient, diagnostic and day care, reflecting and responding to geographical and health system contexts and even using telemedicine as a model of integration with hospitals’ staff to offer specialists outpatient services Their flexible service development, together with their strategic role as integrator of services at a local level, makes CHs appear as a concrete response to the challenging issues of aging population, multimorbidity and limited resources [15].

In Italy, CHs are defined as facilities with a limited number of beds (≤20) managed by nurses and general practitioners or specialists intended for patients with stable clinical conditions, needing care that cannot be provided at home, but not needing the intensity of care of acute hospitals. Patients can be admitted from hospital wards, emergency rooms, homes, or residential care facilities for the elderly [16]. The recommended length of stay in the facilities is three weeks, extendible to six for cases that need particular care. CHs’ beds can be located in acute hospitals, residential facilities or health care homes (*case della salute*), i.e. community-based facilities which offer health and social services to the population residing in the area. Health care homes are managed by the local primary care department and rely on strong collaboration among different professionals to guarantee continuous and holistic care.

Given the diversity of clinical conditions (case mix) of the patients who can be admitted to the CHs, the professional figures (skill mix) involved in these settings are likely to vary based on the population’s needs, context and purpose of the CH [17]. The debate is ongoing on who are the patients most suited to receive care in the CH and consequently who are the professionals most appropriate to obtain the best patient’s outcomes [18]. To date, studies have shown mixed results on the impact of intermediate care services and their organization on patients’ outcomes such as mortality, avoidable adverse events and destination on discharge, but there is growing evidence that these services can improve function in the elderly [19].

Building on a previous descriptive study that presented the characteristics of the patients who are admitted to CHs and providing a general description of this setting of care in the region of Emilia-Romagna, northern Italy [16], we aimed to go further and assess in a multivariable framework to which extent the characteristics or patients and CHs affect health outcomes, in order to identify who can benefit the most from receiving care in CHs and the best skill mix to deliver care in this particular setting, which might represent a milestone of the continuum of care for older and frail people.

## Research Methods

### Study population

This retrospective observational study included all patients residing in Emilia-Romagna discharged in 2017 from the 16 CHs that were in operation at that time in the region. In case of multiple discharges for the same patient, the first episode was retained in the analyses. Data were retrieved from the Regional Informative System of Community Hospitals (*Sistema Informativo Regionale Ospedali di Comunità, SIRCO*), which includes demographics, source of admission, cause of hospitalization, Barthel score on admission and discharge, length of stay, and destination at discharge. In the informative system, each patient is assigned a pseudonymized identification number, which remains the same across all the health care administrative databases of Emilia-Romagna.

To provide an overview of patients’ medical histories, additional clinical information was collected from primary and secondary diagnoses of all acute hospital discharges occurring 3-years prior to the CH admission (source: hospital discharge records). Specifically, we identified 31 conditions based on the Elixhauser method plus 4 conditions drawn from the Charlson index (myocardial infarction [ICD-9-CM codes 410.x, 412], cerebrovascular diseases [362.34, 430.x–438.x], dementia [290.x, 293.x, 294.x, 310.x, 331.0, 331.2] and leukaemia [204.x-208.x]) that were found to be highly prevalent in our study population [20]. We also detected the presence of hip fractures occurring 30-day prior to the index admission (820.x–829.x).

### Characteristics of community hospitals

Data on skill mix and processes of care (i.e., services, facilities and diagnostic assessment provided during CH stay) were collected using an ad hoc survey. The questionnaire contained 34 multiple-choice or yes–no questions divided into 4 categories (general information, clinical activities, nursing activities, rehabilitation activities). Much content of the survey was drawn by the Community Hospital project, which is part of the NHS Benchmarking Network (*https://www.nhsbenchmarking.nhs.uk/*).

The general information section investigated responsible authority, number of beds and location (health care home vs. acute hospital). The clinical activities section investigated medical support (GP vs. specialist), work shifts (i.e., doctors involved on weekdays, weekends, off days and nights) and availability of specialists’ consultations. The nursing activity section investigated the presence of case managers (i.e., nurses who manage the entire clinical pathway of the patients), work shifts for nurses (number of members per shift and weekly working hours). The rehabilitation activity section investigated the presence of physiotherapists, other health professionals and gyms. CH characteristics that were actually included in the analysis for the present study are listed in the Statistical analysis subsection.

The questionnaires were sent by email in May 2018 to the chief medical officers of the local health care authorities and to the hospital trust responsible for the CH, and were collected, always via email, through August 2018. Questionnaires were created and filled using the Microsoft Word Developer tools; collected data were then entered into Microsoft Excel. Thirteen out of the 16 CHs operating at the time in the region responded to the questionnaire.

### Outcome measures

We investigated 3 outcomes measured at the patient level that are expected to be influenced by the skill mix and processes of care of the CHs, as well as by the patient characteristics [10,21,22]. More specifically, the outcomes of interest were:

- Barthel scoring improvement ≥10 at discharge
- Transferring to acute care hospital
- CH length of stay ≥20 days, which corresponds to the length of stay recommended by the Italian law [16].

The Barthel index is a scale that measures a patient’s degree of independence; its score ranges from 0 to 100 and is inversely proportional to the patient’s degree of disability. The SIRCO database contains Shah’s modified version of the index [23]. We dichotomized Barthel scoring improvement in order to express the effect size of its potential predictors with the same summary measures used for the other 2 study outcomes (see Statistical Analysis subsection for further details); for this reason, a sensitivity analysis was performed by using different cut-offs in place of 10.

### Exclusion criteria

Because the clinical presentation and progress of younger patients can differ from those of patients who are typically referred to intermediate care services, subjects aged <65 years were excluded from the analyses. Another exclusion criterion was death during CH stay.

Patients with a score on admission >90 were excluded from the Barthel index analyses, because these subjects could not experience the study outcome (scoring improvement ≥10) on a 0 to 100 scale.

### Statistical analysis

All variables were summarised as means or percentages, as appropriate.

A set of multivariable analyses was carried out to assess the association of the study outcomes with the case mix, skill mix and processes of care of CHs. To estimate the impact of patient- and CH-level predictors and to account for clustering, a two-level random-intercept logistic regression analysis was performed. Due to the limited number of second-level units (the 13 CHs), these models were estimated using the Bayesian Markov chain Monte Carlo (MCMC) instead of the default frequentist approach [24]. Results of the regression analysis are expressed as “posterior” odds ratios (ORs) and 95% “credible” intervals. Interpretation of these quantities does not differ much from interpretation of their frequentist counterparts. To quantify the proportion of total outcome variance that lies at the CH level, we computed the variance partition coefficient (VPC), another quantity that can be obtained from these regression models. The VPC ranges from 0 to 1, and a higher VPC denotes a higher variability in outcomes across CHs [25]. Methodological details about the Bayesian MCMC estimation process used in this study are provided in supplementary Text S1.

To determine the portion of variability across CHs accounted for by patient case mix and organizational variables, three distinct regression models were built for each study outcome. The first model had no covariates (i.e., explanatory variables), the second model included patient-level covariates (case mix), and the third model included both patient- and CH-level covariates (case mix, skill mix and process of care). If the VPC decreases when covariates are added to the model, part of the variation across CHs is accounted for by these variables. To aid VPC interpretation, estimates of CH-specific ORs and their 95% credible intervals resulting from regression models were plotted around the null value of 1, which indicates no difference relative to the overall weighted odds. These estimates arise from the shrinkage estimates of CH-specific random effects, and are related to the ratio of observed-to-expected rates (O/E), which represents the CH risk relative to the overall risk [26].

The CH-level covariates included in these models were medical support (specialist versus general practitioner), years of operation (≥2 vs <2), nurses’ weekly working hours per hospital bed (>12 vs ≤12), and patients seen in a year (≥150 vs <150). The choice of these cut-offs was based on a median split. The other attributes of CHs were constant or heavily skewed, and thus excluded from multivariable analyses. Due to concerns for over-fitting and misclassification, not all patient-level characteristics have been included in the models. A subset of all candidate covariates, including demographics, source of admission, Barthel score on admission, hip fracture, and Elixhauser conditions, was preliminary chosen for inclusion in regression models using an automated selection method which is described in detail elsewhere [27,28]. Scores on admission have been included in all of the Bayesian multilevel models for Barthel improvement, including the first, empty model.

All analyses were carried out using the Stata 15 software (StataCorp. 2017. *Stata Statistical Software: Release 15*. College Station, TX: StataCorp LP).

### Ethics statement

Health care administrative data are pseudonymised on a regular basis at the regional statistical office of Emilia-Romagna, where each patient is assigned a unique identifier that eliminates the ability to trace the patient’s identity or other sensitive data. Pseudonymised administrative data can be used without the approval of an ethical committee and without a specific written informed consent when patient information is collected for health care management and health care quality evaluation and improvement (according to art. 110 on medical, biomedical and epidemiological research, Legislation Decree 101/2018). In Italy, anonymous administrative data-gathering is subject to the law *Protection of individuals and other subjects with regard to the processing of personal data, ACT of 675 of 31.12.1996*.

Patients and the public were not involved in the design or planning of the study. All procedures performed in this study were in accordance with the 1964 Helsinki Declaration and its later amendments.

## Results

### Patient case mix

A total of 3059 patients were discharged from the 16 CHs of Emilia-Romagna during the study period. Of these, 280 (9.2%) were aged <65 years and other 166 (5.4%) died during CH stay, and were excluded from the analyses. Because of missing data, we operated the additional exclusion of 284 (9.3%) patients referring to three non-respondent CHs. See supplementary Table S1 for a comparison of patient characteristics between respondent and non-respondent CHs: patients referring to non-respondent CHs were more independent on admission (mean Barthel score: 42.5 vs. 31.1, *P*-value < 0.001); no other significant differences were observed.

The characteristics of the 2329 patients included in the study, overall and by CH, are summarized in ***Table 1***: except patient demographics, the case mix appeared to be different across the 13 CHs. The distribution of single clinical conditions identified with the Elixhauser method, overall and by CH, is presented in supplementary Table S2. Of note, the 291 (12.5%) patients who experienced multiple admissions to CHs over the study year had a lower prevalence of hip fractures (8.9% versus 20.3%, *P*-value < 0.001) and had more comorbidities (avg. n. per patient = 2.6 versus 2.1, *P*-value = 0.001), as compared to the other patients; no other significant differences in their case mix were found.

**Table 1:** Characteristics of the 2329 study patients, overall and by community hospital, Emilia-Romagna, year 2017.


COMMUNITY HOSPITAL	PATIENTS		% FEMALE	MEAN AGE	MEAN BARTHEL SCORE AT ADM.	% HIP FRACTURES	AVG. NUMBER OF CONDITIONS	% ADM. FROM ACUTE HOSPITAL

	N	%						

1	465	20.0	66.5	81.4	18.7	35.7	2.0	92.9

2	70	3.0	65.7	83.1	31.7	0.0	2.0	0.0

3	24	1.0	70.8	84.7	38.1	4.2	1.7	37.5

4	298	12.8	63.1	83.9	20.4	31.2	2.6	96.0

5	177	7.6	61.6	80.6	23.1	14.7	1.8	68.9

6	201	8.6	65.2	81.9	32.1	18.4	2.0	63.2

7	123	5.3	54.5	83.7	37.2	4.9	3.5	53.7

8	96	4.1	64.6	81.9	40.5	3.1	1.6	46.9

9	33	1.4	66.7	84.4	47.9	0.0	1.9	21.2

10	131	5.6	67.2	83.2	29.8	17.6	2.1	53.4

11	176	7.6	63.1	82.2	33.2	35.8	2.3	90.9

12	119	5.1	49.6	81.7	23.2	12.6	3.2	95.8

13	416	17.9	53.6	82.7	51.8	1.7	1.9	100.0

*All*	*2329*	*100.0*	*61.5*	*82.4*	*31.1*	*18.9*	*2.2*	*79.6*


### Study outcomes

After excluding scores on admission >90 (n = 111), we found 1248 patients with an improvement in Barthel index ≥10 (56.3%). We observed 262 transfers to acute hospital (11.2%) and 1049 CH stays ≥20 days (45.0%); the maximum length of stay in our data was 5 months. The observed CH-specific outcome rates are presented in ***Table 2***.

**Table 2:** Rates of Barthel scoring improvement ≥10, transfer to acute care hospital, and length of stay ≥20 days in 13 community hospitals of Emilia-Romagna, year 2017.


COMMUNITY HOSPITAL	BARTHEL SCORING IMPROVEMENT ≥ 10		TRANSFER TO ACUTE CARE HOSPITAL		LENGTH OF STAY ≥ 20 DAYS	
		
	N	%	N	%	N	%

1	340	73.3	43	9.2	158	34.0

2	3	4.5	8	11.4	49	70.0

3	5	23.8	6	25.0	14	58.3

4	196	66.0	28	9.4	155	52.0

5	79	45.7	15	8.5	94	53.1

6	124	63.3	20	10.0	139	69.2

7	43	38.4	34	27.6	67	54.5

8	57	64.8	20	20.8	54	56.3

9	5	15.6	3	9.1	16	48.5

10	67	55.4	14	10.7	85	64.9

11	116	67.4	22	12.5	99	56.3

12	38	32.8	16	13.4	53	44.5

13	175	48.7	33	7.9	66	15.9

*All*	*1248*	*56.3*	*262*	*11.2*	*1049*	*45.0*


### Skill mix and processes of care of community hospitals

Of the 13 CHs participating in this study, 7 had been operating for ≥2 years (range = 1 to 3.1 years) and 7 admitted ≥150 patients over the study period (range = 24 to 465). The mean number of hospital beds was 17.1 (range = 8 to 20). One CH was led by a teaching hospital, while the other 12 were led by local health care authorities. Nine were located in health care homes, 3 were located in acute hospitals and one was located in a residential facility. Eight CHs were led by GPs and 5 by specialists.

Clinical activity was organized differently across the CHs. Seven had GPs present in the facility on weekday mornings; in 5 of these, GPs were on call in the afternoon. In 5 CHs, care was ensured by specialists; in the smallest one, GPs offered medical consultation only through phone calls. In all CHs, specialists or first-aid physicians guaranteed clinical activity on weekends, off days and nights. Specialist medical advice was available in all CHs when needed.

Nurses’ weekly working hours per hospital bed were on average 12.5 hours (7 CHs ≤12 hours; range = 6.4 to 21 hours). Rehabilitation was delivered by physiotherapists in 12 CHs, and in 11 a gym was present.

### Association of patient case mix with the study outcomes

Results from multilevel regression analyses are presented in ***Table 3***. Hip fracture and admission from acute care hospital were associated with greater improvements in Barthel scores during CH stay; on the contrary, the presence of specific clinical conditions (i.e., metastatic tumours, cerebrovascular diseases and dementia) and older age were associated with lower improvements.

**Table 3:** Results from Bayesian multilevel logistic regression analysis: association of patient case mix with the study outcomes, Emilia-Romagna, year 2017. Odds ratio (OR) estimate is not available (—) if the patient-level predictor was discarded in preliminary automated regression analyses; clinical conditions not shown in the table were also discarded in preliminary analyses.


PREDICTOR	BARTHEL SCORING IMPROVEMENT ≥10		TRANSFER TO ACUTE CARE HOSPITAL		LENGTH OF STAY ≥20 DAYS	
		
	POSTERIOR OR	95% CRED. INTERVAL	POSTERIOR OR	95% CRED. INTERVAL	POSTERIOR OR	95% CRED. INTERVAL

Sex (female vs. male)	1.15	0.94–1.40	0.83	0.63–1.09	1.30*	1.07–1.55

Age (1-year increase)	0.95*	0.94–0.96	0.99	0.97–1.003	0.99	0.98–1.01

Barthel score at admission (1-point increase)	1.02*	1.01–1.02	0.99*	0.98–0.995	0.99*	0.986–0.993

Squared-Barthel score at admission (1-point increase)	0.999*	0.999–0.999	—		—	

Hip fracture	1.65*	1.24–2.16	0.70	0.45–1.01	1.42*	1.09–1.78

Chronic kidney disease	—		1.90*	1.28–2.70	—	

Metastatic cancer	0.45*	0.22–0.83	—		—	

Cerebrovascular diseases	0.69*	0.54–0.86	—		1.45*	1.16–1.80

Dementia	0.61*	0.47–0.79	—		—	

Admission source (hospital vs.home/residential facility)	1.46*	1.08–1.93	—		—	

*Variance Partition Coefficient (VPC)*	*0.271*		*0.062*			*0.147*	


* Significant at the 5% level.

**Figure 1:**
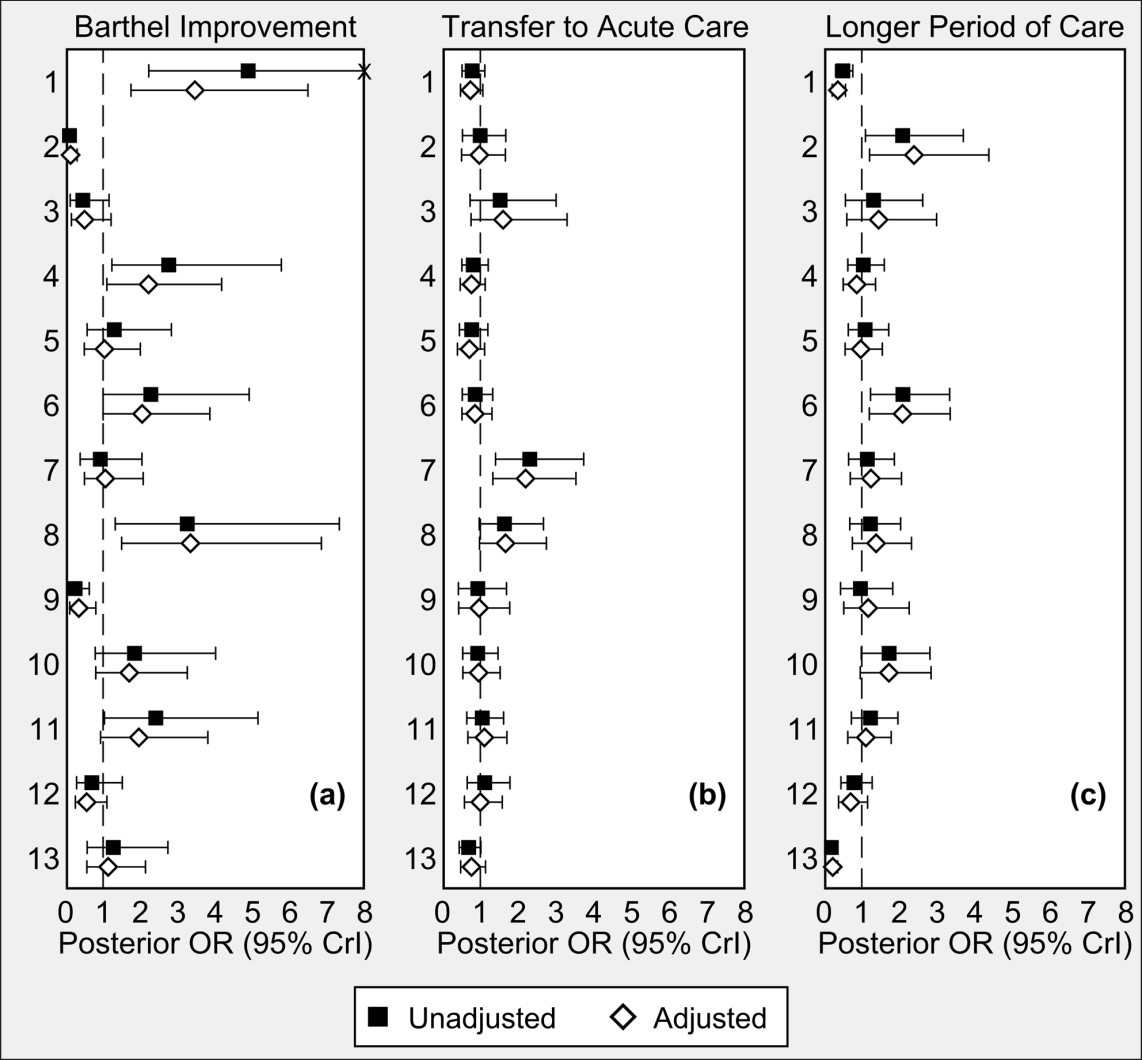
Results from Bayesian multilevel logistic regression models for **(a)** Barthel scoring improvement ≥10 points, **(b)** transfer to acute care hospital, and **(c)** length of stay ≥20 days: CH-specific odds ratios (ORs), unadjusted and adjusted for patient case mix, Emilia-Romagna, year 2017. Dashed line indicates the null value of 1 (no difference compared to the overall average). **Note**: Posterior ORs of Barthel scoring improvement were adjusted for sex, age, squared Barthel score on admission, hip fracture, metastatic cancer, cerebrovascular diseases, dementia, and admission source. Posterior ORs of transfer to acute care were adjusted for sex, age, Barthel score on admission, hip fracture, and chronic kidney disease. Posterior ORs of longer period of care were adjusted for sex, age, Barthel score on admission, hip fracture, and cerebrovascular diseases. The “unadjusted” posterior ORs of Barthel improvement were controlled for squared Barthel score on admission. **Abbreviations**: CrI, credible interval.

As indicated by the linear and quadratic terms of baseline Barthel index, highest and lowest scores had less chances of improvement, compared with patients with a medium level of independence on admission (see supplementary Figure S1). When accounting for the variables shown in ***Table 3*** (see the Statistical Analysis subsection for methodological details), the VPC decreased from 0.331 to 0.271, suggesting that a portion of variability across CHs was explained by the patient case mix (both VPCs are controlled for patient admission scores). As also illustrated in ***Figure 1*** (Panel a), the dispersion of CH-specific random effects around the mean was lower after adjustment for patient characteristics. However, the presence of 5 outliers (i.e., CHs with 95% credible intervals of adjusted ORs including 1) suggests that CH effects still accounted for significant variability in Barthel scoring improvement. When we considered thresholds for Barthel improvement (≥5 to ≥20), the pattern of CH-specific ORs around 1 and the number of outliers did not change appreciably (see supplementary Figure S2).

As shown in ***Table 3***, less independent patients (as indicated by lower Barthel scores on admission) and patients with chronic kidney disease were associated with higher risk of transfers to acute care settings. Less independent patients, hip fractures and cerebrovascular conditions were also associated with longer periods of intermediate care. However, the informative contribution of these covariates to the variability across CHs was of no significance (hospital transfer-VPC 0.059 to 0.062; longer stay-VPC 0.138 to 0.147). It should be noted too that the variability across CHs in these two outcomes was lower than the variability in Barthel improvements (***Figure 1***, Panels b and c).

### Association of skill mix and process of care with the study outcomes

None of the parameters involving skill mix and processes of care were significantly associated with the study outcomes (***Table 4***). Of note, the VPCs resulting from adding these variables to the models were virtually coincident with those from previous analyses (Barthel improvement-VPC: 0.271 to 0.285; hospital transfer-VPC: 0.062 to 0.055; longer stay-VPC: 0.147 to 0.143).

**Table 4:** Results from Bayesian multilevel logistic regression analysis: association of skill mix and care processes of community hospitals with the study outcomes, Emilia-Romagna, year 2017. Odds ratio (OR) estimates are adjusted for patient-level characteristics listed in Table 3.


PREDICTOR	BARTHEL SCORING IMPROVEMENT ≥10		TRANSFER TO ACUTE CARE HOSPITAL		LENGTH OF STAY ≥20 DAYS	
		
	POSTERIOR OR	95% CRED. INTERVAL	POSTERIOR OR	95% CRED. INTERVAL	POSTERIOR OR	95% CRED. INTERVAL

Medical support (specialist vs GP)	1.66	0.28–6.71	0.79	0.45–1.31	0.51	0.15–1.22

Years of operation (≥2 vs <2)	1.16	0.23–3.91	0.95	0.55–1.51	1.24	0.46–2.85

Nurses’ working hours/week/bed (>12 vs ≤12)	0.94	0.14–3.72	1.20	0.70–2.00	1.39	0.47–3.26

Patients/year (≥150 vs <150)	3.78	0.59–13.6	0.61	0.33–1.07	1.02	0.31–2.54

*Variance Partition Coefficient (VPC)*	*0.285*		*0.055*		*0.143*	


## Discussion

Previous studies have shown the efficacy of integrated care services, but it is important to move from efficacy to effectiveness. For this purpose, it is crucial to study in real-world settings which patients can benefit the most from this type of services and which models of care influence the outcomes.

In this population-based observational study conducted in CHs, which can be considered as a prototype of integrated care services, 56.3% of the patients had an improvement in their functional ability during hospitalization (Barthel scoring improvement ≥10). We found that younger age, hip fracture and admission from acute care hospital were associated with greater improvement, while metastatic tumours, cerebrovascular diseases and dementia were associated with lower improvement. According to Dixon et al. [17], less independent patients on admission (i.e., patients with a lower Barthel score) had greater improvement in Barthel scoring; our evidence showed that highest and lowest Barthel scores had less chances of improvement compared with patients that presented with a medium level of independence. A plausible explanation is that low Barthel scores necessitate more intensive care than that offered in CHs, while at the opposite, patients with the highest scores might be admitted to CHs for social reasons that we could not control for in our study.

In our study, 88.8% of the patients were sent home to continue treatment closer to family or in a familiar environment, and only 45.0% had a CH stay ≥20 days. Significant predictors of unplanned transfer to acute hospital were chronic kidney disease and lower independence at admission, while significant predictors of longer length of stay were female sex, hip fracture, cerebrovascular diseases and, again, lower independence at admission. These data suggest that CHs could be the appropriate setting of care for older patients needing rehabilitation or low intensity care, with the purpose of enabling them to experience a better quality of life after discharge from an acute hospital or to prevent them from being hospitalized when not necessary. Other studies too have shown that intermediate care can enable patients to regain abilities in daily living, decrease readmissions and reduce mortality [29,30,31,32,33].

The study patients showed similar demographic characteristics and most of them were elderly with multiple conditions and significant functional decline in the activities of daily living. As other authors have pointed out, resource consumption and poor outcomes are significantly higher for these patients due to their clinical and socio-demographic conditions [34,35]. Our findings too suggest that clinical outcomes are negatively affected by age and multimorbidity, which is more frequent with increasing age [36].

Overall, the case mix was different across the 13 CHs. Indeed, this heterogeneity is in line with the very nature of CHs that, acting as an integrator between primary care services and acute hospitals, can play different roles in different contexts, adapting to the catchment area in which they are implemented and being developed in order to address the local factors and the different needs of the population they serve [15].

The organizational model of CHs, similarly to other intermediate care services, faced this challenge of adapting to the local context and responding to the different needs of the populations by drawing patient centred services (involving an adequate skill mix), therefore building an integrated system not only among different settings, but also among disciplines and professions [15,37]. Concerning skill mix, we reported that clinical responsibilities in the CHs of Emilia-Romagna more frequently involved physicians from various disciplines, contrary to what happens in other countries where usually the doctors involved are GPs [38,39,40,41,42,43]. In 8 out of 13 CHs, GPs had clinical responsibilities; specialists were available for clinical consultation in all the structures, and in 5 of them they also had clinical accountability and were present in the structure at all times. Nurses held great managerial and patient-related tasks, similarly to other countries. [39,44]. To our knowledge, though, there is no clear answer to who should be accountable in order to obtain the best outcomes for the patient. As emerged from our survey, all involved professionals reported working together as a multidisciplinary/multi-professional team. This could be seen as an advantage per se, because collaborative capacity is fundamental for integrated care services. However, developing effective teams and multidisciplinary approaches might be easier said than done [18]: for this purpose, it is necessary to build relationships between partners in care and to establish trust and willingness to take shared accountability for outcomes.

Contrary to our expectations, no statistically significant association was found between CH skill mix and care process with the study outcomes. The implementation of integrated care models involves workforce changes, including new leadership and management roles, new professional roles and new working environments [37], while we know that the age of our 13 CHs was relatively young when the study took place. Accordingly, the growth of competencies to establish sustainable integrated care solutions should start with accurate education and training of health professionals that need to use tools and instruments to support and foster integration on a day-to-day basis [18,37]. In addition, as already pointed out in the literature, it is not easy to measure what happens at the service- and clinical-level in terms of team working. Indeed, our results indicate that in 5 “outlying” CHs (***Figure 1***) a part of the variability in the Barthel scoring improvement could be attributed to specific characteristics of the services that we were not able to detect with our survey. New research on intermediate care, using mixed quantitative-qualitative methods such as the positive deviant approach, could help highlight specific aspects of skill mix, multidisciplinary care and effective team working that impact on the patient’s level of independence. This approach consists in identifying “positive deviants” in health care, which are those organization consistently demonstrating high performance in an area of interest (quantitative approach) in order to deeply investigate which characteristics and models of care differentiate these best performing organizations (qualitative approach) [45].

We argue that another important condition that should be investigated and considered is patient’s frailty, a condition broader than multimorbidity that could highly impact clinical outcomes [46,47,48,49,50]. Frailty is generally considered as a state of increased vulnerability to stressors, characterized by a decline in functioning across multiple physiological systems, associated with increased risk of disability, mortality, hospitalization, falls and admission to long-term care [51,52]. Frailty is also described as a multidimensional condition, with multiple factors contributing to its development, including physical (chronic disease), biological, physiological functional, cognitive, psychological, social and economic aspects. The continuous life-long rising of frailty makes it harder to meet the health and social needs of the elderly [53].

A strength of this study is the use of subject-level data thanks to the availability of a unique patient identifier in the informative systems of Emilia-Romagna. We also created an ad hoc questionnaire with the purpose of investigating the organizational models of CHs, giving us the opportunity to analyse specific features such as nurses’ and support staff’s weekly working hours and specific medical support. Another strength is that the response rate to the survey was relatively high (>80%), although patients referring to non-respondent CHs had higher levels of independence at admission and this might have lowered the overall rates of Barthel score decay, transfer to acute care and longer CH stay in Emilia-Romagna. The main limitation to our study is the small number of CHs investigated, which prevented us from assessing the role of a wide range of potential predictors of health outcomes at the CH level; however, we took all the necessary precautions to deal with the small number of CHs under study, including Bayesian MCMC estimation techniques applied to hierarchical regression modelling. A small population of CHs makes it difficult also to provide an extensive description of their individual characteristics and to present cross-tabulations, which might reveal indirectly the identity of one or more facilities. A second limitation is that in 2017 a standardized field reporting the cause of CH admission was not available in the SIRCO database of Emilia-Romagna, but we tried to surmount this issue by analysing the source of admission (hospital vs. home or residential facility) as a proxy for it. A third limitation is that we did not follow up patients after discharge, so we cannot conclude that favourable study outcomes were associated with a lower risk of hospital readmission or death. Lastly, the Barthel index is a convenient measurement scale to assess physical functional disability but fails to capture changes in functional ability related to general health, communication status and psychological status.

## Conclusions

Our analysis did not reveal a statistically significant association of CH skill mix and care processes with the study outcomes. The presence of some outlying CHs in which Barthel scoring improvement could be attributed to undetected characteristics of the services provided suggest that the various models of intermediate care are difficult to summarise and describe in terms of effectiveness within the limits of a standard quantitative approach. Novel approaches, such as mixed quantitative-qualitative methods, could be used to evaluate thoroughly the outcomes of intermediate care services and, more generally, of integrated care approaches. Increasing awareness of patient frailty (bio-psycho-social conceptual framework) may further foster integrated services to be even more tailored to the specific needs of the patients.

## Data Accessibility Statement

Data can be available if requested.

## Additional files

The additional files for this article can be found as follows:

10.5334/ijic.5566.s1 Supplementary Text. Text S1.

10.5334/ijic.5566.s2 Supplementary Tables. Table S1 and Table S2.

10.5334/ijic.5566.s3 Supplementary Figures. Figure S1 and Figure S2.
