# Supplementary material for: The Contribution of Case Mix, Skill Mix and Care Processes to the Outcomes of Community Hospitals: A Population-Based Observational Study
Source: Int J Integr Care. 2021 Jun 21;21(2):25. doi: 10.5334/ijic.5566 (PMC8231454; doi:10.5334/ijic.5566)
Supplement: Supplementary Text. — Text S1. [file ijic-21-2-5566-s1.pdf]

SUPPLEMENTARY TEXT

Text S1. Technical specification of the Bayesian Markov chain Monte Carlo (MCMC) estimation process in two-level random-intercept logistic regression analysis.

We used zero-mean normal priors for regression coefficients and inverse-gamma prior for the variance of random intercept (i.e., variability across community hospitals). Because prior distributions have an increased effect on posterior distributions when sample sizes are smaller, we attempted an alternate model estimation using a half-Cauchy prior for the variance component, but results did not change appreciably. In each regression model we specified 20,000 MCMC samples with a burn-in period of 5,000 to ensure convergence for all model parameters, and set a thinning interval of 5 to decrease the autocorrelation of the simulated samples. Posterior distributions of regression coefficients, including the shrinkage estimators of hospital-specific random effects, were summarized with exponentiated means (posterior odds ratios) and with 2.5th and 97.5th percentiles (95% credible intervals). The posterior distribution of random-intercept variance was summarized via the variance partition coefficient (VPC). All covariates were mean-centred to mitigate multi-collinearity and to allow for blocking of parameters in MCMC estimation.
