# Supplementary material for: The Contribution of Case Mix, Skill Mix and Care Processes to the Outcomes of Community Hospitals: A Population-Based Observational Study
Source: Int J Integr Care. 2021 Jun 21;21(2):25. doi: 10.5334/ijic.5566 (PMC8231454; doi:10.5334/ijic.5566)
Supplement: Supplementary Tables. — Table S1 and Table S2. [file ijic-21-2-5566-s2.pdf]

SUPPLEMENTARY TABLES

Table S1. Population characteristics in respondent and non-respondent community hospitals of Emilia-Romagna, year 2017.

Patient characteristic	13 respondent community hospitals (*n* = 2329)		3 non-respondent community hospitals (*n* = 284)		*P*-value
	n	%	*n*	%	
Female	1432	61.5	173	60.9	0.852
Age, mean ± SD	82.4 ± 7.6		80.0 ± 7.5		0.110
Barthel score on admission, mean ± SD	31.1 ± 29.1		42.5 ± 30.7		<0.001
Hip fracture	440	18.9	48	16.9	0.416
Number of Elixhauser conditions, mean ± SD *	2.2 ± 2.2		1.8 ± 2.2		0.186
Admitted from acute care hospital	1854	79.6	215	75.7	0.126

* Plus myocardial infarction, cerebrovascular diseases, dementia, and leukaemia.

Abbreviations: SD, standard deviation.

Table S2. Distribution of clinical conditions occurring 3-years prior to the community hospital admission, overall and by community hospital of Emilia-Romagna, year 2017. Conditions with prevalence <1% are not reported.

Clinical condition	Community hospital																											
	1		2		3		4		5		6		7		8		9		10		11		12		13		*All*	
	n	%	*n*	%	*n*	%	*n*	%	*n*	%	*n*	%	*n*	%	*n*	%	*n*	%	*n*	%	*n*	%	*n*	%	*n*	%	*n*	%
Congestive heart failure	57	12.3	14	20.0	5	20.8	68	22.8	23	13.0	20	10.0	39	31.7	17	17.7	6	18.2	25	19.1	31	17.6	24	20.2	58	13.9	*387*	*16.6*
Cardiac arrhythmias	75	16.1	18	25.7	5	20.8	94	31.5	23	13.0	36	17.9	47	38.2	17	17.7	4	12.1	37	28.2	39	22.2	24	20.2	83	20.0	*502*	*21.6*
Valvular disease	25	5.4	2	2.9	2	8.3	29	9.7	4	2.3	13	6.5	8	6.5	4	4.2	0	0.0	4	3.1	9	5.1	9	7.6	13	3.1	*122*	*5.2*
Pulmonary circulation disorders	11	2.4	2	2.9	0	0.0	22	7.4	4	2.3	7	3.5	5	4.1	1	1.0	2	6.1	2	1.5	3	1.7	2	1.7	5	1.2	*66*	*2.8*
Peripheral vascular disease	16	3.4	3	4.3	2	8.3	21	7.0	7	4.0	10	5.0	11	8.9	5	5.2	2	6.1	5	3.8	17	9.7	18	15.1	30	7.2	*147*	*6.3*
Hypertension, uncomplicated	115	24.7	8	11.4	2	8.3	120	40.3	23	13.0	35	17.4	29	23.6	9	9.4	5	15.2	40	30.5	56	31.8	49	41.2	122	29.3	*613*	*26.3*
Hypertension, complicated	63	13.5	6	8.6	3	12.5	17	5.7	27	15.3	25	12.4	42	34.1	6	6.3	6	18.2	17	13.0	31	17.6	9	7.6	18	4.3	*270*	*11.6*
Paralysis	2	0.4	0	0.0	0	0.0	1	0.3	5	2.8	4	2.0	5	4.1	0	0.0	0	0.0	2	1.5	3	1.7	0	0.0	3	0.7	*25*	*1.1*
Other neurological disorders	15	3.2	5	7.1	1	4.2	22	7.4	3	1.7	15	7.5	9	7.3	1	1.0	2	6.1	5	3.8	9	5.1	14	11.8	19	4.6	*120*	*5.2*
Chronic pulmonary disease	49	10.5	14	20.0	1	4.2	39	13.1	17	9.6	11	5.5	20	16.3	7	7.3	2	6.1	13	9.9	14	8.0	26	21.8	54	13.0	*267*	*11.5*
Diabetes w/ complications	59	12.7	10	14.3	3	12.5	56	18.8	23	13.0	24	11.9	19	15.4	8	8.3	4	12.1	24	18.3	21	11.9	32	26.9	54	13.0	*337*	*14.5*
Diabetes w/o complications	19	4.1	3	4.3	0	0.0	7	2.3	7	4.0	6	3.0	3	2.4	1	1.0	1	3.0	4	3.1	5	2.8	9	7.6	9	2.2	*74*	*3.2*
Hypothyroidism	6	1.3	1	1.4	1	4.2	16	5.4	3	1.7	6	3.0	13	10.6	0	0.0	1	3.0	2	1.5	4	2.3	2	1.7	20	4.8	*75*	*3.2*
Chronic kidney disease	43	9.2	3	4.3	1	4.2	37	12.4	13	7.3	21	10.4	26	21.1	7	7.3	4	12.1	5	3.8	19	10.8	14	11.8	31	7.5	*224*	*9.6*
Liver disease	8	1.7	3	4.3	0	0.0	4	1.3	1	0.6	5	2.5	3	2.4	2	2.1	1	3.0	5	3.8	3	1.7	5	4.2	17	4.1	*57*	*2.4*
Metastatic cancer	8	1.7	1	1.4	0	0.0	7	2.3	5	2.8	3	1.5	3	2.4	4	4.2	0	0.0	2	1.5	1	0.6	2	1.7	10	2.4	*46*	*2.0*
Solid tumour w/o metastasis	30	6.5	3	4.3	0	0.0	20	6.7	13	7.3	17	8.5	10	8.1	11	11.5	1	3.0	4	3.1	9	5.1	14	11.8	31	7.5	*163*	*7.0*
Rheumatoid arthritis	4	0.9	0	0.0	0	0.0	5	1.7	3	1.7	5	2.5	4	3.3	0	0.0	2	6.1	7	5.3	4	2.3	4	3.4	6	1.4	*44*	*1.9*
Obesity	5	1.1	0	0.0	2	8.3	3	1.0	5	2.8	5	2.5	5	4.1	0	0.0	1	3.0	2	1.5	3	1.7	8	6.7	9	2.2	*48*	*2.1*
Weigh loss	15	3.2	1	1.4	0	0.0	2	0.7	2	1.1	3	1.5	4	3.3	3	3.1	0	0.0	3	2.3	2	1.1	6	5.0	9	2.2	*50*	*2.1*
Fluid and electrolyte disorders	45	9.7	8	11.4	0	0.0	11	3.7	6	3.4	12	6.0	8	6.5	3	3.1	0	0.0	5	3.8	16	9.1	11	9.2	17	4.1	*142*	*6.1*

Blood loss anaemia	8	1.7	1	1.4	0	0.0	6	2.0	7	4.0	13	6.5	6	4.9	2	2.1	2	6.1	2	1.5	2	1.1	2	1.7	17	4.1	*68*	*2.9*
Deficiency anaemias	8	1.7	1	1.4	2	8.3	7	2.3	5	2.8	9	4.5	9	7.3	3	3.1	0	0.0	3	2.3	7	4.0	3	2.5	21	5.0	*78*	*3.3*
Psychoses	5	1.1	2	2.9	0	0.0	4	1.3	0	0.0	3	1.5	6	4.9	0	0.0	2	6.1	0	0.0	0	0.0	5	4.2	4	1.0	*31*	*1.3*
Depression	15	3.2	4	5.7	0	0.0	18	6.0	4	2.3	10	5.0	7	5.7	2	2.1	2	6.1	4	3.1	7	4.0	11	9.2	15	3.6	*99*	*4.3*
Acute myocardial infarction	9	1.9	2	2.9	1	4.2	17	5.7	6	3.4	3	1.5	7	5.7	2	2.1	1	3.0	8	6.1	5	2.8	8	6.7	10	2.4	*79*	*3.4*
Cerebrovascular disease	135	29.0	13	18.6	5	20.8	45	15.1	42	23.7	45	22.4	38	30.9	23	24.0	7	21.2	26	19.8	42	23.9	31	26.1	56	13.5	*508*	*21.8*
Dementia	66	14.2	10	14.3	4	16.7	67	22.5	41	23.2	34	16.9	31	25.2	9	9.4	6	18.2	18	13.7	41	23.3	35	29.4	33	7.9	*395*	*17.0*
