# Supplementary material for: The Contribution of Case Mix, Skill Mix and Care Processes to the Outcomes of Community Hospitals: A Population-Based Observational Study
Source: Int J Integr Care. 2021 Jun 21;21(2):25. doi: 10.5334/ijic.5566 (PMC8231454; doi:10.5334/ijic.5566)
Supplement: Supplementary Figures. — Figure S1 and Figure S2. [file ijic-21-2-5566-s3.pdf]

SUPPLEMENTARY FIGURE

Figure S1. Results from Bayesian multilevel logistic regression models for Barthel improvement ≥10 points: predicted probability of improvement as a function of Barthel score on admission. Emilia-Romagna, year 2017.

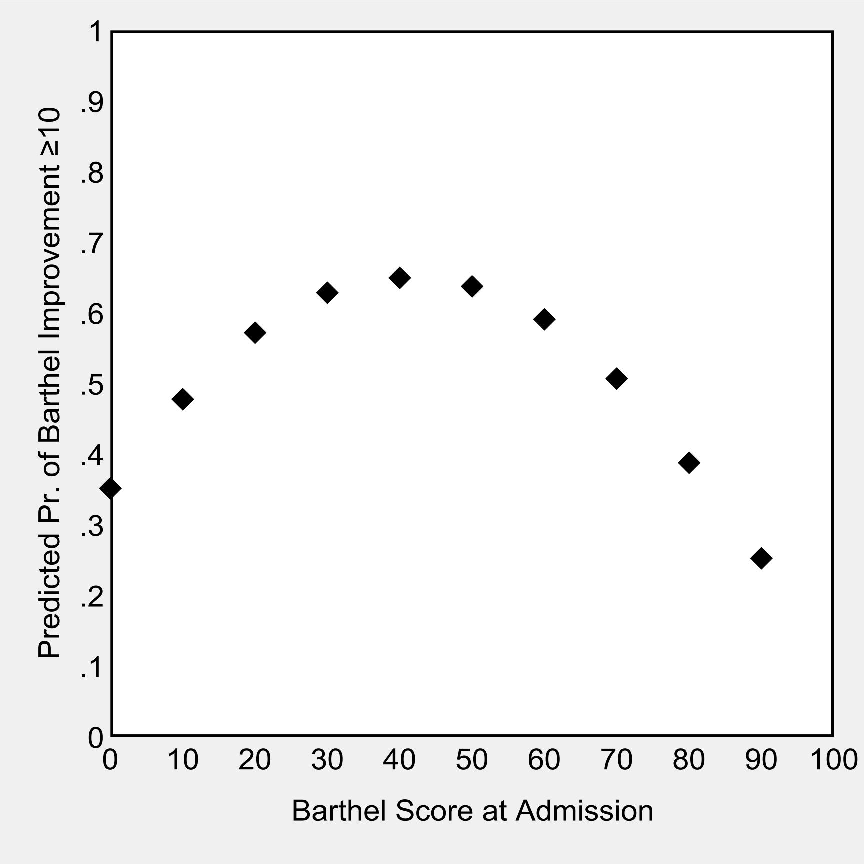

Note: Predicted probabilities were adjusted for sex, age, hip fracture, metastatic cancer, cerebrovascular diseases, dementia, and admission source.

Figure S2. Results from Bayesian multilevel logistic regression models for Barthel scoring improvement (**a**) ≥5 points, (**b**) ≥15 points and (**c**) ≥20 points: CH-specific odds ratios (ORs), unadjusted and adjusted for patient case mix, Emilia-Romagna, year 2017. Dashed line indicates the null value of 1 (no difference compared to the overall average).

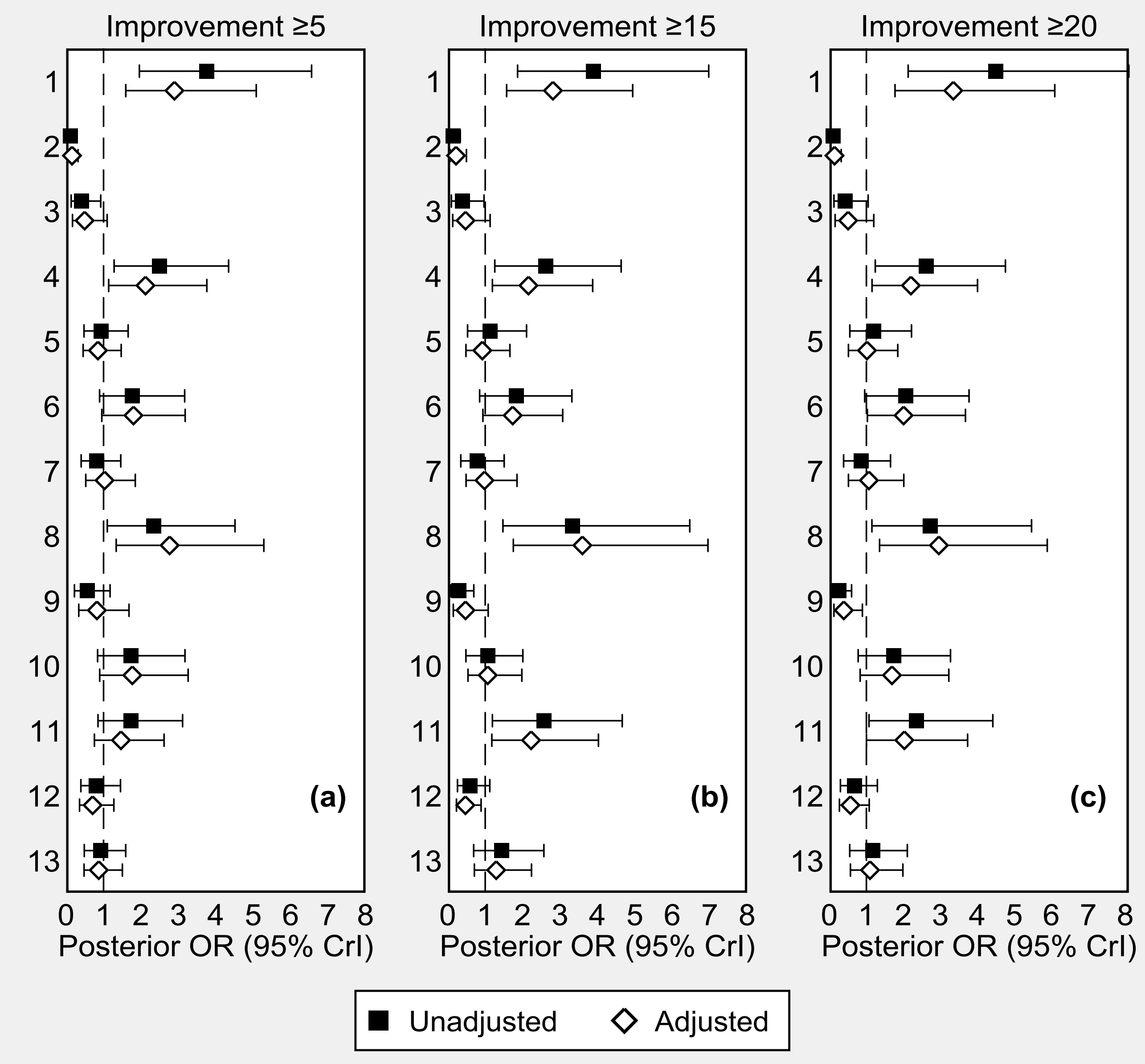

Note: Posterior OR estimates were adjusted for sex, age, squared Barthel score on admission, hip fracture, metastatic cancer, cerebrovascular diseases, dementia, and admission source. The "unadjusted" posterior ORs of Barthel improvement were controlled for squared Barthel score on admission. After adjustment, the variance partition coefficient (VPC) changed from 0.255 to 0.206 for ≥5-scoring improvement, from 0.293 to 0.225 from ≥15-scoring improvement, and from 0.318 to 0.257 for ≥20-scoring improvement.

Abbreviations: CrI, credible interval.
